# Peer Review Quality and Transparency of the Peer-Review Process in Open Access and Subscription Journals

**DOI:** 10.1371/journal.pone.0147913

**Published:** 2016-01-29

**Authors:** Jelte M. Wicherts

**Affiliations:** Department of Methodology and Statistics, Tilburg School of Behavioral Sciences, Tilburg University, Tilburg, The Netherlands; Lancaster University, UNITED KINGDOM

## Abstract

**Background:**

Recent controversies highlighting substandard peer review in Open Access (OA) and traditional (subscription) journals have increased the need for authors, funders, publishers, and institutions to assure quality of peer-review in academic journals. I propose that transparency of the peer-review process may be seen as an indicator of the quality of peer-review, and develop and validate a tool enabling different stakeholders to assess transparency of the peer-review process.

**Methods and Findings:**

Based on editorial guidelines and best practices, I developed a 14-item tool to rate transparency of the peer-review process on the basis of journals’ websites. In Study 1, a random sample of 231 authors of papers in 92 subscription journals in different fields rated transparency of the journals that published their work. Authors’ ratings of the transparency were positively associated with quality of the peer-review process but unrelated to journal’s impact factors. In Study 2, 20 experts on OA publishing assessed the transparency of established (non-OA) journals, OA journals categorized as being published by potential predatory publishers, and journals from the Directory of Open Access Journals (DOAJ). Results show high reliability across items (α = .91) and sufficient reliability across raters. Ratings differentiated the three types of journals well. In Study 3, academic librarians rated a random sample of 140 DOAJ journals and another 54 journals that had received a hoax paper written by Bohannon to test peer-review quality. Journals with higher transparency ratings were less likely to accept the flawed paper and showed higher impact as measured by the h5 index from Google Scholar.

**Conclusions:**

The tool to assess transparency of the peer-review process at academic journals shows promising reliability and validity. The transparency of the peer-review process can be seen as an indicator of peer-review quality allowing the tool to be used to predict academic quality in new journals.

## Introduction

Peer review is a core mechanism for quality control in scientific publishing, but the quality of peer review itself is often obscured by the fact that it takes places behind closed curtains in most journals [1]. Although academic journals need to publish high quality work in order to obtain and maintain a good standing within their particular scientific fields, several recent controversies have highlighted problems with peer review in both traditional (subscription) journals [2] and in Open Access journals [3]. The relation between peer review criteria and the business models used in publishing may differ. For instance, more traditional publishers of multidisciplinary top journals with a subscription format (i.e., readers paying for access to articles) might aim to publish only articles with a large expected readership, and select for novelty and potential impact. On the other hand, publishers working under the gold Open Access (henceforth OA) model, where authors pay a fee to publish their scholarly work, might be more interested in publishing solid work, regardless of potential impact, and will hopefully offer high quality peer review. An increasing number of new peer-reviewed journals publish under the OA model [4–6]. A potential problem related to the quality of peer review that is perhaps specific for the OA publication model is that publishers generate revenue by accepting papers, which may lower the quality of the peer-review if editorial decision-making is insufficiently independent from (short-term) economic considerations [7]. In a recent project, Bohannon [3], submitted a fatally flawed paper for publication to 307 OA journals to have it accepted in 157 of those journals. Although Bohannon’s study was limited to OA journals and hence did not investigate potential similar problems with substandard peer review at traditional subscription journals [8], his results raised questions about the quality of peer-review in some OA journals. Even journals listed in the Directory of Open Access (DOAJ) and/or published by well-known publishers like Sage and Elsevier accepted his flawed paper (while PLOS ONE rejected it). In light of the fact that the peer-review continues to be mostly a closed system [1], substandard peer-reviews may not be easily exposed. Although potentially substandard peer-review systems are certainly not specific to OA journals [2, 8, 9], Bohannon’s results and reviews by Beall [7] do suggest that at least some OA journals have relatively low standards of peer-review. Jeffrey Beall maintains a list of so-called predatory publishers with substandard peer review, but his criteria are often vague and his selection methods have received some criticism [10]. How, then, can we determine whether journals offer solid peer review? In peer-reviews of (past) performance of researchers and research groups and in predicting potential impact of articles and journals, it is customary to consider bibliometric data alongside more qualitative reviews [11–16] and so perhaps impact factors can offer some guidance. However, in light of the novelty of many (OA) journals, and their common lack of an ISI Web of Science impact factor, many new (OA) journals have yet to establish a name in their scientific field. Hence, it has become increasingly important for authors, funding organizations, readers, academic institutions, and publishers to assure the quality of peer-review at young (predominantly OA) journals. Because quality of peer-review is difficult to assess without having peers rate the peer review reports on academic grounds, I focus here on a more generic and accessible aspect of the peer-review, namely the transparency with which the academic journal (and/or its publisher) presents its peer-review process to readers and potential contributors.

The goal of the current project was to develop a questionnaire to assess the transparency of the peer-review process that is (1) easy to use by readers, authors, reviewers, editors, librarians, and other stakeholders (2) transparent in terms of scoring rules, (3) based on criteria that are expected to be acceptable for most stakeholders (authors, reviewers, peers, editors, librarians, publishers, and funding organizations), (4) applicable to a wide range of fields, and (5) composed of a set of items that shows internal consistency. Here I report three studies of the validity and reliability of this transparency tool. Table 1 provides an overview of the studies and the research questions addressed in each study. The first study involved authors of articles published in traditional (originally non-OA) journals and focused on whether author’s ratings of peer-review transparency predicted their assessment of the quality of peer-review at that journal, and whether these variables were related to journal’s impact factor. The second study was conducted during an open-access meeting in which publishers, funders, editors, and other experts of (OA) publishing rated the items of the tool for relevance, and used the tool to rate three types of journals that were expected to differ in terms of transparency and academic standards. In the third study, a group of academic librarians rated the transparency of journals in the Directory of Open Access Journals (DOAJ), which is the largest database of OA journals. The third study also involved the ratings of journals to which Bohannon submitted his hoax paper [3], to see whether rated transparency of the peer-review process could predict the acceptance or rejection of a flawed paper by those journals. The three studies address the core psychometric properties of the transparency tool (internal consistency, dimensionality, and inter-rater reliability) and the validity of the transparency tool as assessed by the impact factor or a related measure of impact (Studies 1 and 3), the degree to which authors rate the quality of the peer review, (Study 1), whether the journals were published by so-called predatory publishers (Study 2), and whether or not the journals accepted Bohannon’s flawed paper (Study 3).

**Table 1:** An overview of the studies of the transparency tool and questions addressed in each study.

Study	Respondents	Targets (what did respondents score?)	Questions addressed	Version
1	235 authors of articles	92 non-OA journals in which they published	internal consistency of the tool	1a
			dimensionality of the tool	
			does transparency predict peer review quality?	
			does transparency predict impact factor?	
2	OA conference attendees	31 journals: 9 OA, 13 traditional, 9 predatory	can transparency predict type of journal?	1a
			inter-rater reliability	
			internal consistency of the tool	
			dimensionality of the tool	
2	20 stakeholders	16 items in the tool	do stakeholders consider the items relevant?	1b
3–1	18 librarians + 2 experts	random sample of 140 DOAJ journals	determine norms for transparency in DOAJ	2a
			internal consistency of the tool	
			dimensionality of the tool	
			does transparency predict h5 impact factor?	
3–2	8 librarians + 2 experts	54 OA journals from Bohannon’s study	can transparency predict rejection of paper?	2a
			internal consistency of the tool	
			dimensionality of the tool	
3	16 librarians	14 items in the revised tool	do librarians consider the items relevant?	2b

Notes: Versions used refer to 1a: the 15 item version in Table 2; 1b: the ratings of relevance of the 16 items in Table 3; 2a: the 14 item version in Table 6; 2b: the ratings of relevance of the 14 items in Table 4.

The underlying idea for developing the tool to rate transparency of the peer review is that transparency is an indicator of a rigorous and accountable system of editorial peer-review, or to put it differently, that opaque peer-review systems facilitate substandard peer-review processes [7]. For instance, if a journal lets editorial assistants employed by the publisher either conduct the reviews or make decisions about acceptance (something that appears to have happened in some of the journals to which Bohannon submitted his paper), economic considerations like increasing revenue may take over academic considerations concerning quality. A transparent peer review system will convey to readers and potential contributors how the peer review is implemented and how articles are selected for publication, thereby rendering it more difficult to keep such an obscure operation from being criticized in the academic community (and beyond). Note that most of my studies focused on OA journals, yet the transparency tool can be used to judge any academic journal exercising peer review.

## Study 1: Authors, Impact Factors, & Peer Review Quality

In a recent survey of biomedical researchers (N = 1,340) at top universities in the world, only 25.1% indicated that the peer-review system in their field was transparent, and slightly less than half indicated it to be fair (48.4%) or scientific (47.5%) [9]. This suggests that peer-review is not considered highly by all (biomedical) researchers and that they consider the transparency of the peer-review process to be low. This raises the possibility that more transparent peer-review systems give rise to higher quality reviews, as assessed by authors.

Peer-review reports often show low inter-reviewer reliability [17], reviewers do not always spot all errors in a manuscript [18], and review quality is often hard to assess [19]. Yet authors’ quality ratings of the review reports of their own work converge with quality ratings by editors of these reviews [20]. Therefore, one straightforward way to assess peer review quality is to ask authors to rate the quality of peer reviews of their own work [21]. The goals of the first study were (1) to see whether author’s ratings of the transparency of the peer review system at the journal where they recently published predicted their assessment of the quality of the peer review of their recently published paper, (2) to assess the internal consistency (reliability) of the tool, (3) to determine whether the items could be reasonably scaled on one dimension (unidimensionality), and (4) to see whether the rated transparency (and ratings of the quality of the peer-review) predicted the journal’s impact factor. Because I needed ISI Web of Science impact factors for all journals, Study 1 only involved traditional (non-OA) journals.

### Method of Study 1

#### Developing the tool

The questions concerning transparency of the peer-review system pertain to information that is accessible on the website of the journal, as part of the journal description, author guidelines, and possibly also in the manuscript submission portal. Given goals 1 (ease of use) and 2 (transparent scoring rule), I chose to use a straightforward five-point Likert scale for all items, even though some items may be answered with yes or no.

Given the dearth of systematic research in this area, my choice for items was based on an informal assessment of information given at websites of a relatively heterogeneous set of journals published by *Elsevier*, *PLoS*, *Sage*, *Frontiers*, *Nature Publishing Group*, and *BioMedCentral*. In addition, I followed guidelines stipulated in the Code of Conduct of the Committee on Publication Ethics (*COPE*) and the Open Access Scholarly Publishers Association (*OASPA*): http://oaspa.org/membership/code-of-conduct/. After receiving and incorporating comments from two independent experts of OA publishing on a earlier version, the final tool (Version 1) contained fifteen items that concern four main themes: explication of criteria during peer-review (Questions 1–3), explication of the peer-review process (Questions 4–6), selection of reviewers/editors (Questions 7–9), publication ethics (Questions 11–12), governance (Questions 10 & 13), and transparency in general (Questions 14–15). A sixteenth item was added that related to whether the member of the editorial board were listed on the website of the journal (this item was not administered in Study 1 because all journals listed the editorial board and hence this item was not expected to show any variance in ratings). The items are given in Table 2. In light of goals 3 (acceptability for majority of stakeholders), 4 (applicability to wide range of journals), and 5 (homogeneity of the scale) I decided against including criteria of transparency that are not (yet) widely accepted across scientific fields, like publishing of reviewer’s names alongside papers. In Studies 2 and 3, various stakeholders were asked to rate the items for relevance.

**Table 2:** Descriptive statistics for items in the original tool in the survey among 235 authors in 92 journals (Study 1).

Item	Loading	M	SD
1. Aims, scope, and expected readership of the journal are clearly specified on the journal’s website	0.41	4.49	0.43
2. Types of submissions that are deemed appropriate for the journal are explicated on the website	0.60	4.30	0.46
3. Criteria used by reviewers to rate submissions are specified on the website	0.52	3.47	0.80
4. The website indicates whether all submissions are sent out for review and who will make final decisions about them (e.g., editor, associate/action editor)	0.63	3.68	0.76
5. The website provides timely updates of the status of submissions during the peer-review process (e.g., under review)	0.64	3.94	0.87
6. The targeted duration of the peer-review process is indicated on the website	0.66	3.48	0.95
7. Authors are allowed to indicate names of non-desired reviewers	0.54	3.26	1.06
8. Reviewers’ names are listed in a yearly acknowledgments to reviewers	0.31	2.95	1.02
9. The identity of the (action/associate) editor who handled a submission is disclosed upon publication	0.52	3.53	1.03
10. Journal discloses the past (yearly) number of submissions, publications, and rejection rates	0.59	3.19	0.76
11. Authors and reviewers are asked to disclose potential conflicts of interest	0.56	3.77	0.84
12. Journal’s website highlights issues of publication ethics (e.g., plagiarism), copyright, and (if applicable) publication fees	0.59	3.92	0.69
13. Published papers include information on dates of original submission and acceptance	0.49	4.32	0.69
14. Website allows ratings of papers and post-publication commentaries by the community	0.40	2.76	0.83
15. Reviewer’s comments and editorial correspondence are published alongside papers	0.49	2.13	0.96
16. Members of the editorial board are listed*	-	-	-

* not administered in this survey because all assessed journals listed their editorial board.

In this study and in the remaining studies, respondents were asked to rate the journal on the applicability of characteristics on a five-point scale in which the points pertain to: 1: “completely fails to apply”, 2: “fails to apply”, 3: “applies partly”, 4: “applies”, and 5: “applies very well”. Respondents were instructed to use the midpoint when the respondent was uncertain about the applicability of a given item.

#### Selection of journals to be rated and author survey

I randomly selected 100 journals from Web of Science from a larger list of journals that were included in a related bibliometric study that was presented in the OA meeting discussed in Study 2. The bibliometric study [22] aimed to predict journal’s impact factor on the basis of citation scores of the members of the editorial boards in these journals. Specifically, for Study 1, I drew a stratified random sample of 20 journals from five main disciplines in the Web of Science database (“Chemistry: Analytical”, “Economics”, “Physics: Condensed Matter”, “Psychiatry” & “Radiology & Nuclear Medicine”), representing diverse fields (albeit without coverage of the arts and humanities). Subsequently, I randomly selected 20 corresponding authors who had recently published a paper in these journals and invited these authors by email to complete an online survey on the LimeSurvey platform on their experiences in the peer-review of their paper. By letting authors rate the transparency of the journal at which they had recently published, I was able to assess whether transparency scores predicted the quality of the peer review as rated by the same authors.

This criterion of transparency for the survey among researchers consisted of the sum of three questions (scored on the same five-point Likert scale as above): ”The peer-review process was fair and transparent”, ”Peer-reviews and editorial comments were rigorous and helpful”, and”I would recommend colleagues to submit their work to this journal”. I expected the criterion to be associated positively with the summed scores on the fifteen items that rated transparency. Although there are different ways to assess quality of peer review [21], I used ratings similar to those used in previous research [20, 23], with the understanding that the current sample only covered accepted papers, so that ratings would probably be on the high side.

In the fall of 2012, I sent out a total of 2006 emails (two selected journals had published 4 or 2 rather than 20 papers and so I added two randomly selected journals within these fields). Of these emails, 224 resulted in automated out-of-office or undelivered mail responses. A further 9 authors indicated by mail that they could not complete the survey (e.g., because their paper was solicited and not peer-reviewed or because of lack of time). One hundred and sixty three authors visited the website but did not complete the online survey and 239 authors completed all questions. I did not send any reminders to non-responding authors because the response rate at the level of the journals (my unit of analysis) was acceptable (>90%). Ethical considerations concerning this study and the other two studies are given in the appendix. Anonymized data and the full questionnaire can be found on the OSF project page: osf.io/5hn36.

### Results of Study 1

Ninety-six journals were rated by at least one author. Four authors provided an incorrect or ambiguous journal name in the survey so were not taken into account in the current analyses (the inclusion of these data did not affect any of the main conclusions given below). The mean sum scores across the remaining 235 respondents was 52.51 (SD = 8.81). I used the eta-squared from a standard ANOVA with journal as sole factor on the basis of individual respondent’s sum scores to estimate the reliability of the mean score per journal (see, e.g., [24]), which provided a reliability of .48 across all disciplines. The reliabilities of the mean overall scores for separate fields were .61, .53, .37, .23, and .46 for Radiology & Nuclear Medicine, Chemistry: Analytical, Psychiatry, Economics, and Physics: Condensed Matter, respectively. These are acceptable reliabilities given the relatively low number of surveyed authors per journal (between 1 and 11; *M* = 2.49, *median* = 2). The remaining analyses were based on scores averaged over authors per journal (i.e., over the 92 journals).

The Alpha reliability of the scale as a measure of the internal consistency of the 15 administered items was computed across all journals (N = 92) and was .81. The Alpha reliabilities for separate fields were: *α* = .82 for Radiology & Nuclear Medicine, *α* = .90 for Chemistry: Analytical, *α* = .81 for Psychiatry, *α* = .62 for Economics, and *α* = .73 for Physics: Condensed Matter. A principal component analysis showed 4 components with Eigenvalues larger than 1. A scree plot showed that there is a dominant 1^st^ principal component (explaining 29.0% of the variance), indicating that it is sensible to compute one sum score reflecting transparency. Descriptive statistics per item and loadings on the first principal component are given in Table 2.

Mean differences across fields on the 15 administered items were non-significant (tested against *α* = .05/15 = .0033), except for Questions 7 and 8. On whether non-desired reviewers could be indicated Economics journals were rated significantly lower than the four remaining fields (after Bonferroni correction at α = .05). On the question related to the acknowledgments for reviewers (Q8), journals in Condensed Matter Physics scored significantly lower than the remaining fields except Chemistry: Analytical (no other pairwise comparisons were significant after Bonferroni correction for these two questions). The sum scores of the fifteen items approached normality and did show some differences between the fields (*F* (4,87) = 3.15, *p* = .018), but differences were fairly small. Bonferroni corrected pair-wise comparisons showed somewhat lower ratings for transparency for Economics journals (M = 49.06, SD = 4.13) as opposed to Radiology & Nuclear Medicine (M = 55.60, SD = 7.45) and Chemistry: Analytic (M = 54.80, SD = 7.01). Psychiatry (M = 52.85, SD = 6.40) and Physics: Condensed Matter (M = 53.60, SD = 5.59) showed averages close to the grand mean (M = 53.19, SD = 6.49).

The 3-item criterion measure had an Alpha reliability (across the 92 journals) of *α* = .86. Averages across journals were quite high on the three items, with “Fairness” receiving M = 4.15, “Rigor” receiving M = 4.22, and “Recommendation” receiving M = 4.34. These averages are somewhat higher than those found in previous ratings of the quality of peer-review [23], supposedly because of the fact that the current survey only covered peer review experiences with a happy outcome for the authors. The correlation was *r* = .48 (*p* < .001) across all 92 journals, and *r* = .66 for Radiology & Nuclear Medicine, *r* = .48 for Chemistry: Analytical, *r* = .60 for Psychiatry, *r* = .44 for Economics, and *r* = .26 for Physics: Condensed Matter. Journals’ Impact Factors (IF) failed to predict both transparency (*r* = .12, *p* = .27) and quality ratings (*r* = .10, *p* = .32). Transparency and quality ratings also showed low and non-significant correlations with the IF within the five fields (all *rs* < .20). Also, there were no mean differences between fields in ratings of peer-review quality (*F* (4,87) = .09, *p* = .98).

### Discussion and Conclusion of Study 1

Study 1 showed that author’s ratings of transparency of the peer-review system at journals in which they published were fairly one-dimensional and sufficiently internally consistent. The ratings predicted author’s assessments of peer-review quality quite well in all five scientific fields. Impact factors failed to correlate significantly with assessments of the quality of peer-review and with ratings of peer-review transparency. Differences between the fields were quite small. At the same time, several items showed relatively low means and little variation in this sample of traditional (non-OA) journals. A limitation of the current study is that it only involved authors whose work had been accepted for publication and who responded to our invitation. Unfortunately, without assistance of journal publishers it is difficult to survey authors who experienced less pleasurable outcomes when submitting their work to the journals at hand. Nevertheless, sampling predominantly those with positive outcomes is expected to lead to some restriction of range on the criterion measure. So the finding of sufficient convergent validity in the current sample does suggest a meaningful relation between author’s assessments of the peer review process and the transparency of the peer review process at the sampled journals.

## Study 2: Stakeholders and different types of journals

The goals of the second study were (1) to see whether different stakeholder’s ratings of the transparency of journals could distinguish between three types of journals: traditional journals, OA journals published by established publishers, and OA journals on Beall’s list of predatory publishers [25–28], (2) to determine inter-rater reliabilities in a sample of journals that was expected to be more heterogeneous in terms of transparency, (3) to again determine internal consistency of the tool, (4) to again determine dimensionality of the tool, and (5) to solicit opinions of various stakeholders on the criteria (items) chosen as indicators of transparency. To these ends, attendees of a two-day conference that concerned the development of quality assessments of new (OA) journals rated a total 31 journals for transparency with Version 1 of the tool, and indicated whether they felt that the criteria (i.e., items) in this tool were relevant. This would enable me to refine the tool and develop Version 2 of the tool that was to be used in Study 3 (see below).

### Method of Study 2

The meeting called “OA vector” was concerned with quality control in OA publishing and took place in Rotterdam, The Netherlands, on 22–23 October 2012 [29]. The invitees were editors, a science reporter, librarians, representatives of national and international funding organizations, and academic publishers. After having seen a presentation of the results of Study 1, the attendees were asked to use the 16 questions to rate journals. The 50 journals were specifically chosen to reflect heterogeneity across scientific fields and publication models, and were randomly distributed over the participants. Thirty-one journals were eventually rated by one or more of the attendees. The rated journals involved 9 journals that were taken from Jeffrey Beall’s list of journals from potential predatory publishers [4]. These journals and publishers were expected to have relatively low standards as explained in some detail by Beal in several writings [25–28]. In addition, participants rated 13 traditional (non-OA) journals from established publishers, and 9 OA journals published by well-known publishers, including *PLoS*, *BMC*, *BMJ*, and *Sage*. Although attendees were aware of the goal of the study, they were not given information on which journals were from Beall’s list. Jeffrey Beall was a participant but did not rate any of the journals from his own list. Also, none of the editors or publishers rated any journal they were associated with.

After the rating of journals, attendees were asked to rate each of the sixteen questions for appropriateness on a five-point Likert scale. Several participants also provided written feedback. Both the ratings and the feedback were completed individually and via an online platform (LimeSurvey). On the second day of the conference, there was an open discussion between attendees on the usefulness of the questionnaire. This open discussion was not recorded to allow qualitative scoring, but a science writer reported on it in *Science* [29].

### Results of Study 2

The three types of papers received clearly different mean ratings: Traditional (non-OA) journals received an average sum of 46.88 (*SD* = 10.68), OA journals published by well-known publishers scored higher on transparency (*M* = 56.41, *SD* = 7.34), while the OA journals published by supposed predatory publishers received the lowest ratings (*M* = 35.72, *SD* = 6.13). Differences were large; Cohen’s d for the comparison between the two types of OA journals was larger than 3. There are two types of dependencies in the data (viz. journals and raters) and so I tested the difference between the three types of journals by running a multi-level regression that included rater and journal as random factors, and type of journal as fixed factor. The difference between the three types of journals turned out to be significant: χ^**2**^ (DF = 2) = 21.25, p < .001. Moreover, I used the model without the type of journal to estimate the inter-rater reliability (i.e., percentage of variance in ratings due to journals) as well as the percentage of variance that could be attributed to raters. In total 52% of the variance could be attributed to journal variance, which translates to a fair intra-class correlation of 0.52. Of the remaining variance, 12% could be attributed to raters.

To determine the internal consistency of the transparency tool, I averaged the ratings across raters for each of the 31 journals. The Alpha reliability equaled .89. The lowest item-rest correlation (r = .04) was found for Item 16, which was the item that was not administered in Study 1 and that referred to the listing of the editorial board members on the journal’s website. All other items had item-rest correlations above r = .37, indicating good psychometric properties. Like in Study 1, a principal components analysis yielded one dominant component explaining 41.9% of the variance. All items’ loadings on this component (except the one for Item 16 which was .04) were above .43, indicating that it is sensible to compute one (unweighted) transparency sum score.

Table 3 provides the results of the “assessment of the assessment”, or the ratings for relevance of the 16 items by the attendees of the OA meeting. As can be seen, more than half of the stakeholders agreed on the inclusion 11 of the 16 items. Results show less support for items 7 (“Authors are allowed to indicate names of non-desired reviewers”), 8 (“Reviewers’ names are listed in a yearly acknowledgments to reviewers”), 9 (“The identity of the (action/associate) editor who handled a submission is disclosed upon publication”), 14 (“Website allows ratings of papers and post-publication commentaries by the community”), and 15 (“Reviewer’s comments and editorial correspondence are published alongside papers”). During the second day of the meeting, the attendees discussed the merits of the scale and although consensus was not achieved on all items (which is perhaps not to be expected given the diverging interests of the different stakeholders), most stakeholders appeared to agree about the need to have a tool to assess transparency [29].

**Table 3:** Descriptive statistics of relevance ratings of the 16 questions in the original tool by 20 stakeholders (Study 2).

Item	%incl.	M	SD
1. Aims, scope, and expected readership of the journal are clearly specified on the journal’s website	95	4.80	0.52
2. Types of submissions that are deemed appropriate for the journal are explicated on the website	90	4.50	0.83
3. Criteria used by reviewers to rate submissions are specified on the website	85	4.40	0.88
4. The website indicates whether all submissions are sent out for review and who will make final decisions about them (e.g., editor, associate/action editor)	85	4.15	0.99
5. The website provides timely updates of the status of submissions during the peer-review process (e.g., under review)	65	3.80	0.70
6. The targeted duration of the peer-review process is indicated on the website	80	3.85	0.67
7. Authors are allowed to indicate names of non-desired reviewers	50	3.55	1.15
8. Reviewers’ names are listed in a yearly acknowledgments to reviewers	45	3.30	0.98
9. The identity of the (action/associate) editor who handled a submission is disclosed upon publication	45	3.30	1.13
10. Journal discloses the past (yearly) number of submissions, publications, and rejection rates	70	4.00	0.92
11. Authors and reviewers are asked to disclose potential conflicts of interest	95	4.55	0.95
12. Journal’s website highlights issues of publication ethics (e.g., plagiarism), copyright, and (if applicable) publication fees	95	4.65	0.93
13. Published papers include information on dates of original submission and acceptance	85	4.25	0.85
14. Website allows ratings of papers and post-publication commentaries by the community	40	3.30	0.98
15. Reviewer’s comments and editorial correspondence are published alongside papers	30	2.90	1.29
16. Members of the editorial board are listed	95	4.80	0.52

%incl. refers to percentage of stakeholders who agreed (4) or agreed strongly (5) with inclusion of the item in the final scale.

### Conclusions of Study 2

So, the results of the application of 16-item scale to three types of journals by the different raters showed promising results. First, the ratings of journal transparency made by the OA conference attendees were able to distinguish the three types of journals well, with Open Access journals published by well-known publishers receiving higher ratings of transparency than traditional (non-OA) journals and OA journals published by potential predatory publishers. Second, the bulk of the variance in ratings was due to differences between journals (52%), with only 12% of the variance being due to raters. In other words, the intra-class correlation as a measure of inter-rater reliability was acceptable at .52. Like in Study 1, the scale showed sufficiently high internal consistency (Alpha = .89), and PCA showed a clear dominant component, with all but one item showing sufficiently high loadings. Third, stakeholders’ assessments of relevance of the 16 items highlighted clear support for 11 of the 16 items, but somewhat lower support for the remaining five items.

## Study 3: Librarians, DOAJ & Bohannon’s Study

I revised the tool on the basis of empirical results from the previous two studies and the discussions and feedback that I received at the Rotterdam meeting. The items of the revised questionnaire (Version 2) are given in Table 4. Items 1, 4, 9, 10, 13, 14, and 15 from Version 1 (see Table 2) were kept. Despite receiving mixed responses among the stakeholders (see Table 3), Items 9, 14, and 15 were kept because they reflect best practices employed by successful open access publishers like PLOS and BMC. After additional feedback from two independent experts of OA publishing (as in the first study), I altered the wording of four items and added questions on the role of the editorial board (Item 13), and data policy (Item 14). Also, some questions were combined to not let the issues tapped in those questions to have too much weight in the final score. Table 4 also includes the ratings of relevance (i.e., the degree to which each item should be included in the transparency assessment) by 16 Dutch academic librarians. As can be seen, the revised tool acquired quite high relevance ratings by the librarians, with most items attaining support.

**Table 4:** The revised tool and descriptive statistics of relevance ratings by 16 Dutch academic librarians (Study 3).

No.	Item	%incl.	M	SD
1	Aims, scope, and expected readership of the journal are clearly specified on the journal’s website (previously #1)	94	4.50	0.82
2	Criteria used by reviewers to rate submissions and types of submissions that are deemed appropriate for the journal are specified on the website	94	4.31	0.60
3	The website indicates whether all submissions are sent out for review and who will make final decisions about them (e.g., editor, associate/action editor) (previously #4)	88	4.00	0.52
4	The website provides targeted duration of the peer-review process and indicates that authors will be updated concerning the status of submissions (e.g., under review)	63	3.88	0.81
5	Authors are allowed to indicate names of (non-)desired reviewers	31	3.13	1.09
6	The identity of the (action/associate) editor who handled a submission is disclosed upon publication (previously #9)	31	2.75	1.12
7	Journal discloses the past (yearly) number of submissions, publications, and rejection rates (previously #10)	75	3.81	0.75
8	Journal’s website highlights issues of publication ethics (e.g., plagiarism), copyright, conflicts of interest, and (if applicable) publication fees	88	4.19	0.66
9	Published papers include information on dates of original submission and acceptance (previously #13)	75	4.13	0.81
10	Website allows ratings of papers and post-publication commentaries by the community (previously #14)	31	3.19	0.83
11	Reviewer’s comments and editorial correspondence are published alongside papers (previously #15)	38	3.06	1.12
12	The names and affiliations of members of the editorial board are listed on the website	100	4.38	0.50
13	The role of members of the editorial board is explicated on the website	63	3.69	0.95
14	The journal has clear guidelines concerning sharing and availability of research data	88	4.25	0.68

%incl. refers to percentage of stakeholders who agreed (4) or agreed strongly (5) with inclusion of the item in the final scale. Previous item rank numbers are given in parentheses.

In this third study, I used the revised tool to assess a large number of OA journals. This time, academic librarians from the Netherlands rated a total 194 OA journals in two phases. The first phase of Study 3 featured a fully random (and hence representative) sample of 140 journals listed in the DOAJ (note that two journals were inadvertently rated twice, but excluding these does not meaningfully chance the results). The distribution of rating scores enables norming of the tool and can help address additional questions related to the psychometric properties and the concurrent validity of the revised tool (Version 2). Specifically, the goals of this first phase of Study 3 were: (1) to get a sense of the distribution of transparency scores in the DOAJ, (2) to check the internal consistency of the revised version of the tool, (3) to determine dimensionality of the revised tool through PCA, and (4) to document the correlation between ratings of transparency (done by academic librarians) and the impact of articles in the journals based on Google Scholar Metrics. The second phase involved 54 journals that did or did not accept Bohannon’s hoax paper [3] for publication. The goal of this second phase was to study the concurrent validity of the tool. Specifically, I wanted to determine whether transparency ratings (made by librarians) could predict this clear indicator of rigor in the peer-review system at the journals. Finally, the data from this second phase again enabled me to corroborate the earlier results concerning the internal consistency and dimensionality of the revised tool.

### Method of Study 3

In the first phase, 18 Dutch academic librarians and two independent experts with knowledge of scientific publishing used the new version to rate a fully random sample of English-language journals in the DOAJ database. The selection of journals was based on the full list of 8617 journals listed in DOAJ on January 31^st^ 2013. From these I first selected the 5733 journals published in English and subsequently drew a simple random sample of 140 journals from that list. Subsequently, the participating librarians were asked to select journals from the list that was uploaded on a spreadsheet on Google Docs. Like in the previous studies, raters were asked to go to the journal website to assess the journal and to provide answers via the LimeSurvey platform. Ratings were conducted in March of 2013. Subsequently, I used the h5-Index from Google Scholar Metrics to study relations with citation performance of articles in each journal. The h5-Index is “the largest number h such that at least h articles in that publication were cited at least h times each” for articles published in the last five years [30]. The h5-Indices were retrieved for 2013, 2014 (until June), and 2015 (until June). Because Google Scholar Metrics only includes journals that have published at least 100 articles in the past five years, not all (young) journals have an h5-Index.

In the second phase of Study 3, a subset of earlier raters were asked to rate a new list of journals using the same survey procedure and the same version of the tool (Version 2), but this time the sample of journals was based on data from Bohannon’s study [3] to see whether transparency ratings could predict whether a journal accepted or rejected the hoax paper. The 54 journals that were rated in this phase were randomly selected from the full set of journals in Bohannon’s study. I selected only active journals (1 journal had turned inactive since the Bohannon study had come out) with a known outcome of rejection or acceptance of the hoax paper. These journals were rated by eight of the librarians from the Dutch Universities and the same two experts. I excluded from this study the journals that were mentioned in the Bohannon article and assured via email that none of the raters had knowledge of the outcome (reject/accept) of Bohannon’s submission at each journal. Although the raters were aware of the purpose of the current study, they were blind to the status (reject/accept) of each article. All ratings were completed within one week after the appearance of Bohannon’s article (from October, 6, 2013 and October, 10, 2013), so that it is fairly safe to assume that most of the publishers had not (yet) changed any information on the journal’s websites in the wake of Bohannon’s publication (this was another reason to exclude titles that were featured in his article).

### Results of Study 3

**Fig 1:**
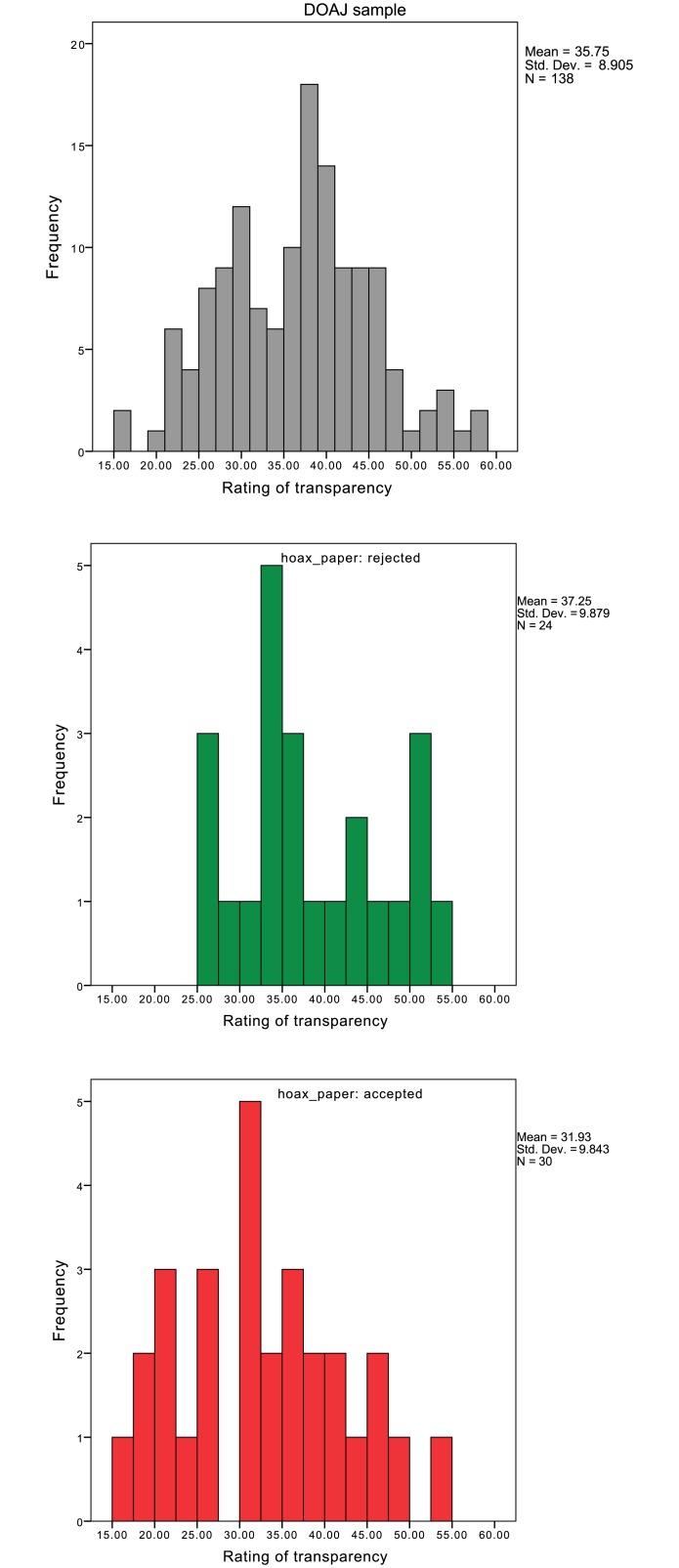
Distributions of transparency scores (sum of all items) in the DOAJ sample (top), and for journals that accepted (middle) or rejected (below) Bohannon’s hoax article.

Distributions of summed ratings of transparency across the 14 items in Version 2 of the tool are given in Fig 1 for the representative DOAJ sample (top panel), for journals that rejected the hoax paper (middle panel), and for journals that accepted the hoax paper (bottom panel). In the random DOAJ sample, the internal consistency of the scale was α = .80. All 14 items showed desirable scale properties, with item-rest correlations varying from .26 for Item 13 to .61 for Item 4. A Principal Component Analysis produced four components with Eigenvalues above 1, with one clearly dominant first component that explained 29.3% of the variance. Like in the earlier studies all (unrotated) loadings on the 1^st^ component exceeded .30, highlighting that the items formed a scale reflecting transparency. Each journal was assessed by only one rater; a multi-level model with rater as random intercept showed that only 5.4% of the variance in ratings could be attributed to raters.

Descriptive statistics for Google Scholar Metrics data and correlations between h5-indices with transparency in the DOAJ sample are given in Table 5. Note that Google Scholar only includes journals that published at least 100 articles in the five-year period. The number of journals that could be found in the Google Scholar Metrics database grew from 31 in 2013 to 55 in 2015. Because in this subset of journals the variance due to raters was very low (0.8% for the 2015 analysis), I analyzed the data in the standard way (i.e., assuming independence). As can be seen, correlations between the transparency ratings and h5-indices were around .30 or even higher. At the same time, whether or not the journals were listed in 2015 could not be significantly predicted by ratings of transparency (p>.20).

**Table 5:** Descriptive statistics for Google Scholar Metrics’ H5-index from 2013, 2014, and 2015 and correlations with transparency in the DOAJ sample (Study 3, phase 1).

Year H5 index	M	(SD)	Median	Skew	N	Correlations with transparency	
						r	r_s_
2013	12.45	(8.27)	11	0.89	31	.394*	.440*
2014	12.35	(8.38)	11	0.96	49	.298*	.298*
2015	12.98	(8.84)	12	1.44	55	.310*	.263+

r_s_: Spearman rank-order correlation;

*p < .05,

^+^p = .052

In the sample of 54 papers that were part of Bohannon’s study (Phase 2), the internal consistency of the scale was α = .90. All items showed good item-rest correlations varying from .44 (for Item 5) to .78 (for item 2). The PCA provided one dominant component, explaining 44.4% of the variance.

I tested the difference between the two types of journals (either accepting the hoax paper or not) by running a logistic mixed-effect regression predicting the acceptance or rejection of the paper on the basis of the sum score as fixed effect with rater as random effect. The sum score significantly predicted the rejection of the paper. Specifically, journals that accepted Bohannon’s paper attained lower overall ratings for transparency (*M* = 31.9, *SD* = 9.8) than journals that rejected it (*M* = 37.3, *SD* = 9.9); Cohen’s *d* = .53. Item-level analyses, using the same multi-level model applied to single items, are given in Table 6, where it can be seen that the items that most strongly predicted rejection of the flawed article by the journals concerned the disclosure of the identity of the (action) editor (Item 6) who handled the submission and publication ethics statements (Item 8) on the journal’s website.

**Table 6:** Descriptive statistics of transparency ratings for DOAJ journals and journals that accepted or rejected the hoax paper (Study 3).

No.	Item	DOAJ sample (N = 140)	Accepted (N = 30)	Rejected (N = 24)	p
1	Aims, scope, and expected readership of the journal are clearly specified on the journal’s website (previously #1)	3.98 (0.86)	3.17 (0.91)	3.67 (1.09)	.064
2	Criteria used by reviewers to rate submissions and types of submissions that are deemed appropriate for the journal are specified on the website	2.74 (1.37)	2.30 (1.15)	2.79 (1.18)	.122
3	The website indicates whether all submissions are sent out for review and who will make final decisions about them (e.g., editor, associate/action editor) (previously #4)	2.99 (1.38)	2.77 (1.28)	3.29 (1.23)	.126
4	The website provides targeted duration of the peer-review process and indicates that authors will be updated concerning the status of submissions (e.g., under review)	2.70 (1.33)	2.37 (1.10)	2.63 (1.38)	.437
5	Authors are allowed to indicate names of (non-)desired reviewers	1.89 (1.29)	2.13 (1.22)	2.17 (1.24)	.920
6	The identity of the (action/associate) editor who handled a submission is disclosed upon publication (previously #9)	1.71 (1.01)	1.70 (0.65)	2.38 (1.24)	.011
7	Journal discloses the past (yearly) number of submissions, publications, and rejection rates (previously #10)	1.70 (1.04)	1.70 (0.92)	1.87 (1.12)	.521
8	Journal’s website highlights issues of publication ethics (e.g., plagiarism), copyright, conflicts of interest, and (if applicable) publication fees	3.15 (1.38)	2.70 (1.18)	3.37 (1.10)	.032
9	Published papers include information on dates of original submission and acceptance (previously #13)	2.64 (1.65)	1.93 (1.08)	2.79 (1.50)	.017
10	Website allows ratings of papers and post-publication commentaries by the community (previously #14)	1.61 (0.87)	1.50 (0.51)	1.96 (1.08)	.040
11	Reviewer’s comments and editorial correspondence are published alongside papers (previously #15)	1.46 (0.76)	1.50 (0.51)	1.54 (0.72)	.800
12	The names and affiliations of members of the editorial board are listed on the website	4.11 (0.97)	3.63 (0.89)	3.79 (1.10)	.551
13	The role of members of the editorial board is explicated on the website	2.91 (1.23)	2.37 (1.10)	2.62 (1.17)	.398
14	The journal has clear guidelines concerning sharing and availability of research data	2.16 (1.24)	2.17 (1.21)	2.38 (1.17)	.518
	Sum score	35.76 (8.86)	31.93 (9.84)	37.25 (9.88)	.049

p values based on item’s or scale’s prediction of acceptance or rejection of the hoax paper in mixed effect logistic regression.

### Conclusions of Study 3

In both phases of this third study, I found the revised tool to show high internal consistency and one dominant underlying component. This corroborates findings of Studies 1 and 2 and shows that the tool psychometric properties. The variance in ratings that could be attributed to raters in Phase 1 of this study was only 5%, as compared to 12% in Study 2, arguably because raters were more homogeneous and more experienced in rating in the third study. Ratings of transparency in the DOAJ sample showed meaningful positive correlations with the h5-Indices from Google Scholar Metrics, although it should be noted that the h5-Index was not available for the majority of the (new) OA journals in the sample. Also, like any measure of impact, the h5-Index is fairly heterogeneous with respect to (size of the) field and is in part a function of the number of articles published in each journal in the five-year period. Insofar that the h5-Index measures impact and sustainability of the journal, the correlations found in Table 5 do lend further support for the predictive validity of the tool to assess transparency.

The rejection of Bohannon’s hoax paper can be seen as a fairly objective measure of the quality of the peer review process in journals. Of interest here are the comparisons between the three distributions in Fig 1, showing that OA journals that accepted Bohannon’s hoax paper were rated considerably lower on transparency of the peer review system than journals that rejected his paper. Also interesting to note is that the random (and hence representative) sample of DOAJ journals showed transparency ratings that were intermediate between the journals that did or did not accept the hoax paper. This suggests that the DOAJ as of early 2013 included a substantial number of journals that are rated lowly on transparency and that many of these journals can be improved in this regard. In the meantime, the DOAJ has started a re-evaluation of its criteria.

## General Discussion

Attaining high standards of peer review is important for all types of academic journals. Controversial cases of poor review have been documented in many fields and even in the most prestigious (non-OA) journals [1], and in 2014 several well-known publishers had to retract 120 articles because of a peer-review scam in non-OA journals [8]. Similarly, some recent results suggest that at least *some* OA journals appear to value quantity over quality when selecting articles for publication [3, 7]. Such results stress the need to be able to predict the quality of peer review on the basis of available information for both OA and subscription journals. I argued that the transparency of the peer review system at journals can be considered an indicator of the quality of peer review and presented results based on different raters (authors, stakeholders, and librarians) of different types of journals (both non-OA and OA) that were in line with this view.

Study 1 showed that authors’ assessment of the quality of peer review of their own paper could be predicted by the authors’ assessment of the transparency of the peer review process at the journals that published those papers. Although such assessments of quality are inherently subjective, such ratings of peer review quality have previously been used [20, 23], and align well with the core notion that indeed academic peers are able to judge quality. A drawback of the first study was that it involved the same raters of the predictor and criterion and so it would be interesting to see in future work whether similar levels of (predictive validity and) convergent validity could be obtained when using independent sources for the ratings (e.g., librarians rating the journals vs. authors rating peer review quality). Such effort, however, would require sufficiently large samples to be able to deal with the fact that ratings of peer review quality may show substantial variation across authors and papers even in the same journal. Another limitation of Study 1 was that it included only authors whose work had been accepted by the journal they rated and that the response rate at the level of authors was low. This raises questions about potential sampling bias, as is often the case in surveys about peer review among researchers (where response rates are often low [31]). Future research should attempt to include a more comprehensive sample of authors, although such an effort would probably require assistance of the journal publishers to help contact authors whose work was rejected by the journal. Nonetheless, the finding that ratings of transparency predicted ratings of peer review quality even in a potentially range-restricted sample does lend support to the notion that transparency and quality are related.

The second study involved the use of selected groups of journals that differed in terms of expected standards, specifically those published by supposedly predatory publishers and those published by highly regarded publishers (including BMC and PLOS). In this second study, attendees of a meeting on quality of OA journals rated the transparency of different types of journals. Results showed that indeed ratings of transparency could predict well whether Beall [25–28] had labeled the journals as being predatory. Moreover, the tool showed sufficient reliability both internally and across the raters. A drawback of the second study was that raters were aware of the goals of the study and may have guessed on the basis of additional information (e.g., the number of articles appearing in the journal) other than transparency whether the journal held high standards. A clearer understanding of such issues requires further research. Importantly, in this study, different stakeholders supported the inclusion of most of the proposed items in the tool. Nonetheless, feedback received during the OA meeting led me to refine the tool further, leading to Version 2, which had 14 items (see Table 4).

The third study involved independent raters who worked at academic libraries (and two independent experts) and highlighted support for the relevance of most items in the revised tool (Version 2). In addition, like in Studies 1 and 2, the (revised) tool showed good internal consistency and one dominant component reflecting transparency. More importantly, the second phase of the third study involved a relatively objective (if not “golden”) standard of peer review quality based on Bohannon’s work, and showed that outcome-blind ratings of peer review transparency at the journals predicted well which journals would accept a deeply flawed paper for publication. Although this third study involved a random sample from Bohannon’s study, a drawback of his study was that Bohannon’s original sample was chosen rather haphazardly, and that it did not involve a control group of non-OA journals as a control group [8]. At the same time, studies like Bohannon’s are challenging from a legal and ethical perspective (specifically, they entail deceit of editors and reviewers) and so his study did provide a unique opportunity to put the tool to the test, with clear results that supported the validity of the transparency tool.

Established journal’s standing within a scientific field is often reflected in their impact factor or alternative metrics of impact. Yet such metrics are typically not (yet) available for young (OA) journals. In Study 1, journal’s impact factors did not meaningfully predict author’s ratings of transparency. In the DOAJ sample in Study 3 I used the 2013, 2014, and 2015 h5-Indices from Google Scholar Metrics, and found that librarians’ ratings of transparency predicted this measure of sustainability and impact of the journals. Although it should be kept in mind that most OA journals are not yet included in this database, this results does further support the predictive validity of the new transparency tool. The reasons why impact factors were not associated with transparency ratings in the author’s survey are unclear, but perhaps this is due to transparency simply being more important for novel OA journals than for established non-OA journals that already have had considerable time to build a reputation.

Taken together the results of the three studies showed that the (revised) tool has sufficient internal consistency and is sufficiently uni-dimensional for its purposes to assess transparency. All three studies supported the validity of the tool. Although more work would certainly be valuable to validate the tool further (which is a given for all tests), the current results highlight its current usefulness in predicting important outcomes like the authors’ assessments of the quality of the peer review of their papers, and whether or not journals accepted a fatally flawed article. Although the publication of peer review reports could in principle enable peer review of the peer review as perhaps the most optimal control mechanism of the quality of peer review [1], such practices remain uncommon (but see some of the BMC journals). Without readers being able to read the peer review reports themselves, the need for being able to predict peer review standards will remain. I have argued that one option to do this is to consider transparency of the peer review system.

The current results should not be taken to mean that a transparent peer review system guarantees high quality or that the lack thereof necessarily means poor standards, but rather that transparency predicts quality of peer review to a practically useful degree. This is also evident from Fig 1, where even journals that were rated highly on peer review transparency happily accepted Bohannon’s hoax paper (some even without exercising any traceable form of peer review). Also, low standard journals could use the list of criteria in the tool to change how they present their peer review system, while keeping low thresholds for publishing papers. However, the manner in which journals present their review system does enlarge accountability in editorial work, and so it is my expectation that even then transparency could help improve academic quality control.

Discussions among stakeholders and ratings of the relevance of each of items in the tool (see Tables 3 and 4) showed support for including most items, but also some diverging opinions. Given the many different ways in which peer review is practiced, the different interests involved (e.g., academic quality, getting busy researchers to do pro bono reviewing, and various social and economic realities), and the relatively little systematic research into which peer review practices work best, such differences in opinion are to be expected. Notwithstanding this lack of consensus on the relevance of all items, the results did show that the items correlated highly among each other and that most predicted the acceptance of the hoax paper in the correct direction (see Table 6), thereby highlighting their potential usefulness. The best practices as reflected in the items were inspired by practices at various publishers and journals, most of which have not been subject of systematic study. The current results align with the notion that transparency of peer review helps safeguard high standards of peer review in both non-OA and OA journals.

The development of the current tool was inspired by a need among research funders, researchers, librarians, readers, and publishers to get some sense of the quality of new journals. The tool could be applied to any journal that exercises peer review and although complete consensus among stakeholders on the specifics of the tool will be hard to obtain, it is my hope that stakeholders could use it to get some sense of the quality of peer review at journals. Therefore I am glad that to announce that the tool has recently been implemented online by a working group of librarians and the SURF foundation on the following website: https://www.qoam.eu/.

## Appendix

### Ethical considerations (all studies)

Participation of authors (Study 1), attendees at the OA meeting (Study 2), and librarians (Study 3) was entirely voluntary. I indicated in the email to authors (Study 1) that their individual answers would remain confidential. According to Standard 8.05 of the Ethical principles of Psychologists and Code of Conduct of the American Psychological Association, informed consent can be dispensed in such cases as long as anonymity is assured and participants are not expected to have any professional risk attached to their participation. Authors (Study 1) and librarians (Study 3) are not expected to have any conflict of interest in rating the journals. Study 2 involved several editors and employees of academic publishers, but none of them rated a journal they were affiliated with.
